# The Royal Commission on Vaccination

**Published:** 1896-09-12

**Authors:** 


### MEDICAL PROGRESS AND HOSPITAL CLINICS.

[*The Editor will be glad to receive offers of co-operation and contributions from members of the profession. All letters should be addressed to* THE EDITOR, AT THE OFFICE, 428, STRAND, LONDON, W.C.]

### THE ROYAL COMMISSION ON VACCINATION.

### ITS FINAL REPORT FULLY ANALYSED AND CONSIDERED.

We have purposely abstained from making more than a mere allusion to the issue of the final report of the Royal Commission on Vaccination until the document was actually in our hands. It is sufficiently obvious to anyone who reads the *résumés* which have from time to time been published by various journals that some of them have been founded on very imperfect information; and it has appeared to us to be not only the wisest, but the most dignified, course, to wait, especially in view of the importance which is sure to attach in the public mind to the pronouncement of a medical journal on what is, after all, at its root a purely medical matter. Now, however, with the report lying open before us, we hope to be able to give a more complete review of its contents than has yet appeared in the medical press. The points which were laid before the Royal Commission for investigation were as follows:—

1. The effect of vaccination in reducing the prevalence of, and mortality from, small-pox.
2. What means, other then vaccination, can be used for diminishing the prevalence of small-pox; and how far such means could be relied on in place of vaccination.
3. The objections made to vaccination on the ground of injurious effects alleged to result therefrom; and the nature and extent of any injurious effects which do, in fact, so result.
4. Whether any, and if so, what, means should be adopted for preventing or lessening the ill effects, if any, resulting from vaccination; and whether, and, if so, by what means, vaccination with animal vaccine should be further facilitated as a part of public vaccination.
5. Whether any alteration should be made in the arrangements and proceedings for securing the performance of vaccination, and, in particular, in the provisions of the Vaccination Acts with respect to prosecution for non-compliance with the law.

The first thing which strikes one, both in the evidence and in the report, is the large amount of labour which has been expended in historical research. This took two directions: (*a*) The early history of vaccination as introduced by Jenner, and of the various lymphs which were originally and have since been used for purposes of vaccination, and (*b*) the history of small-pox in this century as compared with its history and its prevalence in preceding times, and of the various circumstances, such as the rise and fall of inoculation and the gradual rise of sanitation, which might be supposed to have had a bearing on the great diminution which history shows to have taken place during the earlier part of this century in the prevalence of the disease.

This, however, we may fairly be excused for passing over. Great as is the interest of the historical portion of the report, one cannot but feel that the real question to which an answer is wanted is in regard to the efficacy and safety of modern vaccination. Sir John Simon expressed this succinctly in his evidence when he said, "When I look at the question of vaccination I look at it independently of the question of origins. I look at current vaccination, such vaccination as you will find at any well-conducted vaccination station in England," and it is to this "current vaccination," irrespective of theories of origin, that the recommendations of the report apply.

Coming then to modern times, and to the question of the influence of vaccination upon the mortality of small-pox, we find a careful and exhaustive review of the statistics gathered in regard to six separate towns which had been visited by epidemics of small-pox, Sheffield, Dewsbury, Leicester, London, Warrington, and Gloucester.

Much interest attaches to these statistics in view of the assertions so commonly made by the opponents of vaccination that the whole question is a mere statistic problem.

Comparing the death-rate from small-pox as it has affected the vaccinated and the unvaccinated respectively the following points come out.

In Sheffield we find that—

"At all ages, of 4,151 vaccinated persons in the enumerated population of Sheffield attacked by small-pox up to the date of the census, 200, or 4·8 per cent., died of that disease. Of 552 unvaccinated persons in the enumerated population attacked by small-pox up to the same date, 274, or 49·6 per cent., died of the disease, so that, relatively to their number, and apart from any question of age, for each individual vaccinated person suffering from a fatal attack of small-pox, 10·3 unvaccinated persons were fatally attacked by that disease.

"Of 353 vaccinated children under 10 years of age in the enumerated population attacked by small-pox up to the same date, 6, or 1·7 per cent., died of the disease. Of 228 unvaccinated children attacked by small-pox up to the same date, 100, or 43·9 per cent., died of the disease, so that, relatively to their numbers, for each individual vaccinated child from 0-10 years of age suffering from a fatal attack of small-pox, 25·8 unvaccinated children were fatally attacked by that disease."

Thus it seems clear, so far as gross statistics in one outbreak will tell anything, that an attack of small-pox in a vaccinated person is vastly less fatal than in a person who is unvaccinated, and that the protection afforded is far greater in the early years after vaccination than at a later period of life.

Taking the six towns together—

"The unvaccinated attacked amounted to 2,297; of these 819 died, or 35·6 per cent. 1,433 of those attacked were under 10 years of age; of these 520 died, or 36·2 per cent.; 864 were over 10 years of age, of whom 299 died, or 34·6 per cent. . . . Let us now turn to the mass of cases from which these were selected. It consisted of 11,036 attacks, resulting in 1,280 deaths, *i.e.*, a percentage of 11·5. The cases selected from these as unvaccinated were 2,297, or 20·8 per cent. of the total number, a large sample, in fact, drawn from the bulk. Deducting from the total numbers the attacks and deaths of those said to be unvaccinated, the result is as follows: 2,297 attacks and 819 deaths, or 35·6 per cent. mortality, as we have seen in the class said to be unvaccinated, and 8,739 attacks with 461 deaths, or 5·2 per cent. fatality in the rest of the population attacked by the disease. How is this to be accounted for?"

The case of children under ten years of age is still more remarkable, the fatality among the unvaccinated patients attacked by small-pox being 36·2 per cent., while among the vaccinated it only amounted to 2·7 per cent.

Upon the hypothesis then that vaccination has no relation to small-pox, and no tendency to mitigate the effect of the disease, we have before us an arbitrary selection which might just as well have been made by drawing lots of 20·8 per cent. of the total number attacked; why should those thus selected display so remarkably different a proportion of fatal cases, a death rate to attacks of 35·6 per cent. in the one class and 5·2 per cent. in the other? What reason is there why there should be any substantial difference between the two classes?

Taking child life alone, that is, children between one and ten years of age, the following table is given:—

| VACCINATED. | | UNVACCINATED. |
|---|---|---|
| Attacks, 569; deaths, 16. | ... | Attacks, 1,223; deaths, 380. |
| Fatality, 2·8 per cent. | ... | Fatality, 31·0 per cent. |

The contrast, it is said, is all the more striking when it is remembered that all the doubtful cases are included in the vaccinated class, though many of them had in all probability never been successfully vaccinated; for the doubtful cases in London alone account for six out of the fifteen deaths, and raise the fatality from 1·8 to 2·8 per cent.

"Is the different fatality manifested in the two classes, into which the children between the ages of one and ten years who were attacked by small-pox are thus divided, according as they were or were not vaccinated, a mere freak of chance? It is scarcely possible to believe that it can be so. When it is found that the same contrast is exhibited on comparing the death-rate of the classes of vaccinated and unvaccinated in each of the six towns with which we have been dealing, and even in different districts of Sheffield and Dewsbury, where a similar discrimination between the vaccinated and unvaccinated was made, and when it is borne in mind that both classes lived in the same towns, were of similar ages, and suffered in the same epidemics, it is impossible to believe that there was nothing to distinguish the two classes from one another. . . . The conclusion then is surely irresistible that some circumstance must have existed distinguishing the class selected as vaccinated from that selected as unvaccinated, and which rendered it less liable to suffer fatally from small-pox. The only condition which regulated the distribution of the cases into the one class or the other was the presence or absence of vaccination."

The report then gives statistics of the same import derived from an examination of 10,403 cases at the Homerton Hospital between the years 1873 and 1884, and also of other cases at the Fulham Hospital, and then proceeds to examine into the validity of certain criticisms which have been made of these statistics, and of certain alternative explanations which have been put forward of the results they show.

It has been urged against these statistics that, even though every effort were made to classify the cases correctly, the classification was still open to error, inasmuch as persons might be brought to the hospital with the eruption of confluent small-pox upon them, which would prevent the marks even of efficient vaccination being visible. In regard to this, the Commissioners say it is true that it might be so in some cases, but that both Dr. Gayton and Mr. Sweeting assert that it could have happened very rarely. They do not think that it could make such a difference as to modify substantially the contrast exhibited in the death rate of the vaccinated and unvaccinated classes. They further show that if these doubtful cases, that is, cases who though said to be vaccinated showed no marks, are placed in a separate class, the death rate of this class is considerably higher than the fatality in the vaccinated, but considerably lower than in the unvaccinated class, and that the same result works out in regard to each hospital whose statistics are thus examined.

Again, it has been objected that the statistics given show a much higher death rate in proportion to the attacks amongst the unvaccinated class than was shown by records of small-pox mortality prior to the introduction of vaccination. This, the report says, is no doubt generally true, but it is pointed out that the statistics as to the fatality of small-pox before the introduction of vaccination cannot be relied upon, because then, as is the case at the present time, there was a great difference in the fatality of the different epidemics. "At all events, the fact, or alleged fact, on which the criticism is founded cannot be regarded as a proof that the classification into vaccinated and unvaccinated has not been accurately carried out," so that it does not touch the statistics gathered from the six towns above mentioned.

Some, however, who admit the great contrast between the fatality of small-pox in the vaccinated and the unvaccinated attempt to explain it away by saying that "the unvaccinated are mostly to be found in the poorer and more neglected classes of the population, who would on that account be constitutionally weaker, and less able to resist an attack of small-pox, and to escape a fatal result. Speaking generally, this may be to some extent true, though it is not so at all times and in all places. There are facts stated in the reports we have so often quoted, especially those relating to Warrington, Dewsbury, Leicester, and Sheffield, and in the evidence relating to the last-named town, which seem to show that the explanation suggested cannot be the correct one. In the report on the Warrington epidemic . . . it is expressly stated that the vaccinated and unvaccinated were of the same class, and lived in the same houses and in the same manner. Moreover, the persons admitted into the Homerton and Fulham Hospitals were for the most part, whether vaccinated or unvaccinated, of the pauper class or of the class immediately above it. It is not conceivable that in this section of the population the presence of vaccination or its absence should indicate so marked a difference of constitutional strength as to account for the difference of small-pox fatality which we are now considering."

In regard to the objection which has been raised that the unvaccinated class includes those whose vaccinations have been postponed for medical reasons, and would therefore include a larger proportion of children of delicate constitution, who would be more likely to succumb to an illness, it is said that, after giving due weight to these objections, it is impossible to believe that the cause suggested could account to any material extent for the very large difference which has been shown to exist between the death rate of children under 10 observed in the classes of vaccinated and unvaccinated. "A broad margin might be allowed for error without the force of the argument derived from the contrast being seriously diminished."

Passing now from the question of the influence of vaccination in diminishing the fatality of small-pox in those who are attacked by it, the question is considered how far vaccination has a protective effect against an attack of the disease, and it is shown that, even after making all allowance for a possible transfer of cases from the unvaccinated to the vaccinated class in the course of an epidemic, the incidence of small-pox was more than four times as great on the unvaccinated than upon the vaccinated portion of the community. In regard to this question it is pointed out that a difficulty arises at the outset, from the fact that, when an epidemic of small-pox visits a town, the liability to infection of the inhabitants of different parts of the town may differ widely. But taking as the basis of their calculation the occurrence of small-pox among the inhabitants of houses which had already been invaded by the disease, it was found both at Sheffield, Warrington, Dewsbury, Leicester, and Gloucester, that the proportion of those attacked by small-pox was far higher among the unvaccinated than among the vaccinated.

After considering the various objections which have been raised to the obvious teaching of the statistics given, the Commissioners say: "We think, taking it altogether, that the evidence bearing upon the question whether the vaccinated are less liable to be attacked by small-pox than the unvaccinated points to two conclusions—first, that there is, taking all ages together, less liability to attack among the vaccinated than among the unvaccinated; and next, that the advantage in this respect enjoyed by vaccinated children under ten years of age is greatly in excess of that enjoyed at a more advanced period of life."

Statistics are then quoted from the evidence to show that, besides causing a diminished fatality among those attacked and a diminished liability to attack, vaccination also very favourably modifies the type of the disease, and that in proportion as vaccination is thoroughly done its protective power is increased.

In regard to the efficiency of vaccination as a protection against small-pox, the Commissioners draw attention to the remarkable relationship which is proved to exist between the fatality, the attack rate, and the type of the disease on the one hand, and the fact of being vaccinated or unvaccinated on the other. In their statistics they had had to deal with the same classes of people among the vaccinated and the unvaccinated; these people were differentiated from each other merely by the fact of having been vaccinated, and they ask, if this is a mere arbitrary division, and if vaccination has no protective power, how does it happen that the one class is less liable to suffer fatally from small-pox, less liable to be attacked by it, and when attacked has the disease in a milder type than the other? "But this is not all; we have to ask further how it happens that, whether fatality, attack rate, or type be regarded, the difference between the two classes is much more marked in the case of children under ten years of age, who are nearer the period of vaccination, than it is in the case of those of more advanced years? To these questions those who deny that there is any efficacy in vaccination have furnished no satisfactory answers. If, on the other hand, it be conceded that there is virtue in vaccination, and that it renders the vaccinated less liable to be attacked, or to suffer severely from, or to die of the disease than the unvaccinated, the phenomena are all explained and the difficulty vanishes."

The report then goes carefully into the question of re-vaccination, discussing the question in the light of information gathered from towns which have been attacked by small-pox, and in regard to bodies of people, such as soldiers, sailors, police, postmen, telegraph messengers, and others, either specially protected by vaccination or specially exposed to infection, and sums up thus: "Summing up then the evidence on the subject of re-vaccination, so far as regards this country, we find that particular classes within the community, amongst whom re-vaccination has prevailed to an exceptional degree, have exhibited a position of quite exceptional advantage in relation to small-pox, although these classes have in many cases been subject to exceptional risk of contagion."

In answer to the first point referred to them, the Commissioners say: "It has appeared to us impossible to resist the conclusion that vaccination has a protective effect in relation to small-pox," and give the following as their conclusions:—

"We think:—

"1. That it diminishes the liability to be attacked by the disease.

"2. That it modifies the character of the disease, and renders it (*a*) less fatal, and (*b*) of a milder or less severe type.

"3. That the protection it affords against attacks of the disease is greatest during the years immediately succeeding the operation of vaccination. It is impossible to fix with precision the length of this period of highest protection. Though not in all cases the same, if a period is to be fixed, it might, we think, fairly be said to cover in general a period of nine or ten years.

"4. That after the lapse of the period of highest protective potency the efficacy of vaccination to protect against attacks rapidly diminishes, but that it is still considerable in the next quinquennium, and possibly never altogether ceases.

"5. That its power to modify the character of the disease is also greatest in the period in which its power to protect from attack is greatest, but that its power thus to modify the disease does not diminish as rapidly as its protective influence against attacks, and its efficacy during the later periods of life to modify the disease is still very considerable.

"6. That re-vaccination restores the protection which lapse of time has diminished, but the evidence shows that this protection again diminishes, and that to ensure the highest degree of protection which vaccination can give, the operation should be at intervals repeated.

"7. That the beneficial effects of vaccination are most experienced by those in whose case it has been most thorough. We think it may fairly be concluded that where the vaccine matter is inserted in three or four places, it is more effectual than when introduced into one or two places only; and that if the vaccination marks are of an area of half an inch they indicate a better state of protection than if their area be at all considerably below this."

In regard to the injuries which are stated to arise from vaccination, it is pointed out that the admission that some risk attaches to the operation, an admission which must without hesitation be made, does not necessarily afford an argument of any cogency against the practice, if its consequences be on the whole beneficial and important.

The charges brought against vaccination are, (*a*) that to its mischievous influence is to be traced an increase in the number of deaths from certain specified diseases, of which increase it is, in fact, the cause; and (*b*) that in particular cases injury or death has resulted from the operation.

The following are the diseases an increase in which is said to correspond with the spread of vaccination: Tabes mesenterica, diarrhœa, bronchitis, pyæmia, skin disease, syphilis, convulsions, cholera, diphtheria, pneumonia, atrophy and debility, whooping-cough, erysipelas, scrofula.

Now it appears that although some of these show an increasing mortality, the mortality from others of them is diminishing, and it is asked, Why should vaccination be credited with the increase of those diseases which have increased, and not equally be credited with the decrease where the mortality has diminished?

It is also pointed out that vaccination, in the vast majority of cases, takes place in infancy, and that, if it is the parent of other diseases, and has substantially increased the mortality due to them, we should expect to see some effect produced on infantile mortality as a whole. Yet it is clear that the mortality of infants in the first year of life, as measured by the proportion of their deaths to births, has not increased at all during the times when infant vaccination has been increasing.

Dealing with specific diseases, the case of syphilis is taken first, and it is shown that while it is a fact that during the past twenty years there has been an increase in the deaths due to syphilis in infants under one year of age, the records show that this disease is most largely fatal within the first three months of life—that is, before vaccination has had any opportunity of coming into play, and that, so far as statistics go, they indicate that the increase also has been greatest in that portion of the first year of life which would be practically unaffected by vaccination. It is also stated that, however much syphilis among infants may have increased in England and Wales or in Scotland, it has largely diminished in Ireland during recent years, which "certainly affords strong proof that in Ireland vaccination has not been influential in increasing the prevalence of the disease in question." Curiously enough the statistics from Leicester are brought up to show that, at any rate in certain places, the increase in syphilis is independent of the practice of vaccination; for while during late years there has been a great decrease, amounting to a practical disuse, of vaccination in that town; the increase in the infant mortality from syphilis has been no less than 69·3 per cent., as against an increase of 24·7 per cent. for the whole of England and Wales, which conclusively rebuts the argument of those who seek to connect the increase of mortality from syphilis with the practice of vaccination.

Next as to cancer. Many explanations of the apparent increase in the prevalence of cancer have been given, but it is well known that the fact of such increase is disputed by many people. The report, however, says: "It may be that, in addition to the apparent increase, there has been some real increase in the mortality from cancer, but there is not a shadow of evidence to connect this with the practice of vaccination, whilst there is, as we have shown, evidence pointing the other way."

In regard to erysipelas, it is shown that this disease is chiefly fatal in infants in the first three months of life before it would be likely to be influenced by vaccination, and that its fatality rises and falls in correspondence with the mortality from puerperal fever, which suggests another explanation of its cause. Finally, parliamentary returns afford no evidence of any increase in the mortality from erysipelas. On the whole, they show a diminishing mortality from that disease among the infant population.

In regard to diarrhœa, dysentery, bronchitis, and general infant mortality, the case of Leicester is again quoted as showing that in regard to these diseases that town has not gained, as compared with the rest of the country, by its avoidance of vaccination, and the Report concludes this portion of the subject thus: "Upon the whole, then, we think that the evidence is overwhelming to show that in the case of some of the diseases referred to vaccination cannot have produced any effect upon the mortality from them, and that it has not in the case of any one of them increased the mortality to a substantial, we might even say to an appreciable, extent."

A large number of cases were investigated in which injurious results were alleged to have followed on the performance of vaccination, chiefly erysipelas and syphilis.

In regard to the former it is pointed out that, quite apart from vaccination, there is nothing remarkable in the occurrence of erysipelas in the case of an infant; that it may be caused by scratches and injuries of a most trivial description; and it is shown that when it followed vaccination there were other contributory causes. Nevertheless it is clear that, be these causes what they may, erysipelas, in many of the cases in which it took place, would not have occurred but for the vaccination. In fact, it is admitted that, although the cases in which the virus is conveyed at the time of vaccination are rare, yet that its conveyance has "in some instances been clearly established, the immediate occurrence of erysipelas in several co-vaccinees making it practically certain that some virus was conveyed at the time of the operation."

The Report goes on to say that although it may be hoped by additional care to diminish the number of these cases, yet "a vaccination wound is, like one from any other cause, so long as it exists, a source of some risk."

Besides meeting with cases of inflamed arms and erysipelas, vaccinators sometimes meet with instances of severe eruption attended by fever which may end in death. "These cases may be placed in two groups, one in which the vaccination sores proceed normally but a general eruption, possibly gangrenous, occurs, and a second in which the pocks inflame, and are attended by satellites, and a more limited eruption, possibly due only to external contagion, is produced. Of the first only a single example is to be found in the reports before us, but of the second there have been several." The greater care now exercised in vaccination seems to have reduced these occurrences of late years to a very small number indeed; "still it has not been found possible wholly to prevent them."

In regard to syphilis and its conveyance by vaccination it is said, "The fact that this was possible had been fully established and was generally acknowledged by the medical profession before we commenced our inquiries," but that, although it was known to be possible, "no hint has been given," in the evidence, "of the occurrence of any recent series of vaccination-syphilis cases in British practice."

Among the 279 deaths referred to vaccination during the period 1886-1891, five were attributed to syphilis, but the Commissioners state that they are satisfied that "in none of the five cases is there sufficient evidence to show that the death resulted by syphilis caused from vaccination."

In 26 non-fatal cases, syphilis was alleged to have possibly been communicated by vaccination. One of these could not be traced. "The remaining 25, however, were carefully investigated on our behalf, some by Dr. Barlow, some by Dr. Acland, and 15 of them by those gentlemen jointly." Of these cases, 24 were not cases of syphilis at all. "In the remaining case (case 141) it appears that there was some ground for the allegation, though it is by no means proved that syphilis was communicated by vaccination, or even that the case was one of syphilis at all."

Finally, the Report says: "The evidence offered to us would lead to the belief that whilst with ordinary care the risk of communication of syphilis in the practice of arm to arm vaccination can for the most part be avoided, no degree of caution can confer an absolute security."

In regard to the question of how to lessen the evil effects of vaccination, the Commissioners would put the use of calf lymph in the forefront, because this would afford an absolute security against the communication of syphilis. They state that they think that parents should not be required to submit their children to vaccination by means of any but calf lymph, and that they have no hesitation in recommending that steps should be taken to secure that calf lymph for the purpose of vaccination should be placed within the reach of all, but they leave open the question whether the duty of providing calf lymph should be undertaken by the Local Government Boards in the several parts of the United Kingdom, or whether some other method would be more advantageous.

They have also come to the conclusion that it would be well to extend the age period within which vaccination must be performed in England, Wales, and Ireland, to six months from the date of birth, as it is already fixed in Scotland; and they think it might be advantageously extended even to a year from birth if some security could be obtained that in case of small-pox occurring in a sanitary district the children within the compulsory age should be vaccinated.

They deprecate the practice of vaccinating children within a few days of their birth, as is done at some workhouse infirmaries and lying-in hospitals, unless there be obvious danger of small-pox infection; and if in any case it should be thought necessary, they say that the infant should be kept under observation until the arm is healed, and the operation should be limietd to one insertion. The Local Government Board should settle a suitable form for issue to parents, containing clear and simple rules for guidance in the care of the vaccinated arm. The arm also should be inspected three or four days after the operation as well as later on, and another inspection should be obligatory in the third week. The Commissioners also say: "If children were vaccinated and inspected, as a rule, at their own homes instead of being brought to a public station, we believe the risk of injury would be sensibly lessened."

They think it would be an advantage if postponement were allowed, not only on account of the condition of the child, but for conditions affecting its surroundings, and if provision were made for removal of the child elsewhere, whilst the vaccination wound was unhealed, if the home surroundings were insanitary. They think the vaccination vesicles should not be opened unless for some adequate reason, and that vaccine should be stored in tubes rather than on "dry points." Mention is made of the recent experiments made by Dr. Copeman as to the effect of the storage of vaccine lymph in glycerine; on this no opinion is expressed, but in any case they think each tube should only contain sufficient for the vaccination of one person.

"No instrument should be used for the operation which has not been boiled or otherwise sterilised for the purpose; and the simpler the instrument the better."

If summoned by the parent on account of unfavourable symptoms prior to the time fixed for inspection, the vaccinator should be bound to attend; and the fee to be received by the vaccinator should be adequate to cover all these duties, and if medical attendance should be required it should be the duty of the vaccinator to make such attendance, for which he should receive a fee. In answer to the question as to what other means than vaccination can be used for diminishing the prevalence of small-pox, the Commissioners give a full account of the measures which have been taken to control its spread, and of the way in which they have broken down in various places, and say that "a complete system of notification of the disease, accompanied by an immediate hospital isolation of the persons attacked, together with a careful supervision, or, if possible, isolation for sixteen days of those who had been in immediate contact with them, could not but be of very high value in diminishing the prevalence of small-pox." But as to such means taking the place of vaccination, they say the experiment has never been tried, and, after discussing the various bearings of the question, they further say: "We can see nothing, then, to warrant the conclusion that in this country vaccination might safely be abandoned, and replaced by a system of isolation."

In regard to the exercise of compulsion, the Commissioners say—

"After careful consideration and much study of the subject, we have arrived at the conclusion that it would conduce to increased vaccination if a scheme could be devised which would preclude the attempt (so often a vain one) to compel those who are honestly opposed to the practice to submit their children to vaccination, and, at the same time, leave the law to operate, as at present, to prevent children remaining unvaccinated owing to the neglect or indifference of the parent. When we speak of an honest opposition to the practice, we intend to confine our remarks to cases in which the objection is to the operation itself, and to exclude cases in which the objection arises merely from an indisposition to incur the trouble involved. We do not think such a scheme impossible.

"It must, of course, be a necessary condition of a scheme of this description that it should be such as would prevent an objection to the practice being alleged merely as an excuse to save the trouble connected with the vaccination of a child. We may give the following as examples of the methods which might be adopted. It might be provided that if a parent attended before the local authority and satisfied them that he entertained such an objection, no proceedings should be taken against him. Or, again, a statutory declaration to that effect before anyone now authorised to take such declaration, or some other specified official or officials, might be made a bar to proceedings. We do not think it would be any real gain to parents who had no conviction that the vaccination of their children was calculated to do mischief to take either of these steps rather than submit them to the operation. . . .

"We are quite conscious that the proposal we have made will be regarded by some as a retrograde step in the cause of vaccination. We do not believe that it would prove to be so in practice. . . . At the same time we think it would be well to make the change a temporary one in the first instance, say for a period of five years, and that in the meantime its effects should be carefully watched."

They have no hesitation in recommending the Scotch system as in some respects superior to that prevailing in other parts of the United Kingdom, and say:—

"Whatever diversity of opinion there may be on the point just discussed, there can be no doubt that every effort should be made to remove the causes which now render vaccination burdensome and tend to its discouragement, and . . . . we have no hesitation in expressing the opinion that the Scotch system is in some respects, to which we have called attention, superior to that prevailing in the other parts of the United Kingdom. Its great merit lies in this—that the defaulters are sought out at their own homes by the official vaccinator, and then and there vaccinated by him, unless the parent objects or circumstances render postponement desirable. . . . We think that where a certificate of successful vaccination is not received within the prescribed time a notice should be served upon the parent that a public vaccinator will attend on a day named for the purpose of vaccinating the child, unless the operation has been already performed, and that the only offence rendering the parent liable to prosecution should be the refusal to permit the child to be vaccinated by the public vaccinator when he attends for that purpose. The adoption of such a scheme would render the burden much less than it is where the child has to be taken to a public station, not only for the purpose of vaccination, but again at the end of a week for inspection. The vaccination and inspection would both take place at home. It is not only by rendering vaccination less burdensome that this would be an improvement, it would also, as we have already pointed out, tend to diminish risks of injury which arise when a child is conveyed twice to a public station, and which would be absent if the vaccination and inspection always took place at home. Moreover, the vaccination would be more certain to be postponed when the condition of the child or local or family circumstances make this course desirable. This again would diminish the risk attending vaccination. Further, the proposed change would render it easier to introduce the requirement, already alluded to, that the progress of the vaccination should be more carefully watched than it is at present, and any mistakes made in the treatment of the vaccination vesicles would be likely to be more early discovered and more readily remedied."

In regard to payment of vaccination fees, they say:—

"We think it would tend to promote vaccination if every duly qualified medical man who vaccinated a child successfully were entitled to the fee which is now paid to the public vaccinator. We are aware that there is evidence tending to show that in England and Wales vaccination has been, speaking generally, more efficiently performed by public vaccinators than by other medical men. But the experience of Scotland, where only a minority of the vaccinated are dealt with by official vaccinators, shows that such a system may be a satisfactory one. Safeguards might be provided against inefficient vaccination. The fee should only be allowed in cases where the certificate showed that the child had been vaccinated in accordance with the rules prescribed by the Local Government Board, and if every duly qualified practitioner who vaccinated a child successfully could claim the appointed fee it could properly, and ought, we think, to be made a condition that all children so vaccinated should be liable to inspection, and that the fee should not be allowed when the vaccination did not appear to have been performed in accordance with the prescribed rules. It would not, of course, be necessary to make such an inspection in every case—a limited number of test cases would suffice. The liability to inspection would prevent abuse, and under proper regulation we think the system might be an improvement on any at present existing."

As to the use of calf lymph their opinion is that the State is bound to see that a supply of it is within the reach of every vaccinator.

The vaccinator ought to be under an obligation to afford medical attendance, without cost to the parent, in all cases in which the vaccination does not run an ordinary course, and owing to supervening illness such attendance becomes necessary. Whether the fee paid in respect of vaccination should be fixed at such an amount as to cover this extra attendance, or whether it should be the subject of special compensation is a detail which is left open.

In regard to re-vaccination, they attach great importance to it, but see many difficulties in the way of making it compulsory. At the same time they think it should be in every way encouraged, and that steps should be taken to impress on parents the importance of having their children re-vaccinated not later than at the age of twelve years, and they suggest that an adequate fee should be allowed for every case of successful re-vaccination, by whatever medical man it is performed, and that when small-pox shows signs of becoming epidemic special facilities should be afforded both for vaccination and re-vaccination, and notices should be served on people in the neighbourhood urging their importance.

Notification of small-pox should be everywhere compulsory.

In the meantime, pending legislation, they recommend that persons committed to prison for non-payment of penalties imposed under the vaccination laws should no longer be treated as criminals.

### NOTE ON NASAL AFFECTIONS ASSOCIATED WITH VARIOUS SYSTEMIC DISEASES.

By Patrick Watson Williams, M.D., Lond., Physician-in-Charge of the Department for Diseases of the Throat at the Bristol Royal Infirmary.

The influence of the nasal passages in modifying the condition of the inspired air before it reaches the lungs, while undoubtedly recognised in some measure even by the general public, is of far greater consequence for the continuance of a perfectly healthy state of the lungs and bronchi than is generally believed. Not less important is it to bear in mind that the nasal passages are in many senses only an upward extension of the lower air passages, and that in pathological states in which the trachea and bronchi suffer, the nasal passages are prone to be concurrently involved.

In the first place, I would urge a due recognition of the physiological function of the nasal passages in warming and moistening the inspired air before reaching the larynx. It has been proved by various observers, notably Bloch, Greville Macdonald, and Kayser, that (1) whatever the temperature of the atmosphere, the air, after ordinary inspiration through the normal nasal passages, is always raised to the temperature of the blood before reaching the pharynx; (2) the air, after passing through the nose, is invariably completely saturated with moisture. These important modifications in the characters of inspired air are brought about by the peculiar structure of the lining mucosa, which contains, especially in the region of the turbinated bodies, a rich supply of venous sinuses and lymph spaces. Owing to the arrangement of unstriped muscular fibres the turbinated bodies are more or less erectile, and in a cold or dry atmosphere are distended with blood, the transudation of watery fluid being thereby increased, while the air passing through the walls of the nasal passages is rapidly warmed. On the other hand in a warm or moist atmosphere the turbinated bodies are less distended, and the air not requiring to be raised through so many degress of warmth or moisture to reach the required physiological state.

Yet another fact deserves more wide recognition, viz., that the air after passing through the nasal passages is sterile. This sterility of the inspired air and of the nasal secretions has been attributed to suppositious microbicidal properties of nasal mucus; but St. Clair Thompson and Hewlett have shown that there are no such antiseptic properties in the nasal secretions, and that the sterility of the air, after passage through the nose, is due to the arrest of micro-organisms, and their elimination by the constant movement of the cilia which are found on the mucous membrane of the lower two-thirds of the walls of the nasal passages. They have shown that the few microbes that are not arrested by the vibrissæ of the vestibule are disposed of by this simple physiological mechanism.

The air which is inspired through the mouth is less completely warmed and moistened, and is not rendered sterile before reaching the larynx. Many of the cases of so-called simple pharyngitis and laryngitis hitherto supposed to be due to cold are, probably, in reality due to various micro-organisms, which are enabled to set up the characteristic clinical conditions by infecting the air passages when rendered more susceptible from the effect of the relatively cold air. In this way many cases of recurrent bronchitis and pharyngitis arise. That acute rheumatism is an infectious disease dependent on a micro-organism is now widely conceded, and we have only to recall the fact that acute tonsilitis and acute rheumatism are so intimately associated from a clinical standpoint that there would appear little room for doubt that they are usually dependent on one and the same micro-organism or forms of micro-organisms. If this be so, it is evident that it is more or less an accident whether the tonsils are the seat of local infection or whether the infecting micro-organism enters the general system and acute rheumatic fever results, and thus nasal inspiration is prophylactic against attacks both of tonsilitis and acute rheumatism.

Gouty patients are prone to suffer from acute laryngitis and pharyngitis after exposure to cold, and in these cases there is reason to believe that while the apparently simple inflammatory trouble may be associated with and dependent on micro-organisms, there is in addition the local effect of uric acid displaying itself in the inflamed and therefore injured larynx or pharynx. Nevertheless, the fact that these attacks are most frequently immediately due to exposure to a chill, renders those who suffer from impeded nasal respiration more susceptible. It is hardly necessary to adduce evidence in support of the statement that in asthmatics the inspiration of imperfectly warmed air must tend to set up attacks of this affection.

It is not my purpose to discuss the various causes of nasal obstruction, or their treatment, but I would enter a protest against the dangerous practice of relieving such obstruction by the wholly unscientific and very unnecessary destruction of large areas of these important physiological structures of the nasal passages, a point to which Emil Mager has quite recently directed attention. He remarks* that "by far the most frequent abnormalities in the anterior nares that laryngologists are called upon to treat are the diseased conditions of an obstructive nature, and their aim must be to remove these in order to procure free access of air into the posterior nares. For this purpose various mechanical devices have been used, and it is in the selection of these that much care should be taken in order that the least injury possible to the mucous membrane shall occur, for it has been shown how necessary that membrane is to the economy, and if the removal of any portion of the membrane may be avoided it should certainly be done. That the removal of healthy mucous membrane is often followed by very disagreeable results can hardly be gainsaid. We can do so well with other means than the use of the saw, electric burr, or plane, that, except for such absolute obstructions as require opening (as in diseases of the accessory sinuses), the electric burr should be discarded altogether. The electric saw, plane, or shaver are horrible devices, and can work an enormous amount of mischief. The galvano-cautery is at best a mere temporising device, rarely curing anything, but chiefly destroying the mucous membrane, and thus rendering it powerless for its chief physiological functions. This applies mainly to all conditions about the septum. When the mucous membrane itself is already diseased and redundant, as over the turbinates, the use of the galvano-cautery is of some value. . . . The only instance where the removal of the mucous membrane is not apt to be followed by distressing symptoms is where it is redundant, as over the turbinated bodies, and here the same procedure may be followed as in other mucous membranes." Such statements are strong, but, in the face of the proposals that have been made to remove the greater portion of turbinated bodies, and of the practice of extensively destroying the nasal mucosa by the galvano-cautery, a word of caution and a plea for less radical yet more efficient means is not out of place.

*N.Y. Med. Journ., June 13th, 1896.

*(To be continued.)*

